# Consumer Priorities for Upper-Extremity Outcome Assessments

**DOI:** 10.1016/j.arrct.2026.100663

**Published:** 2026-06-02

**Authors:** Lauren J. Christie, Laura Jolliffe, Brian A. Beh, Clive Kempson, Madeleine J. Smith, Ben Schelfhaut, Natasha A. Lannin

**Affiliations:** a Allied Health Research Unit, St Vincent’s Health Network Sydney, Darlinghurst, New South Wales.; b School of Allied Health, Faculty of Health Sciences, Australian Catholic University, North Sydney, New South Wales.; c Nursing Research Institute, St Vincent’s Health Network Sydney, St Vincent’s Hospital Melbourne, Australian Catholic University, Sydney, New South Wales.; d Allied Health, Peninsula University Hospital, Peninsula Care Group, Bayside Health, Victoria.; e Department of Occupational Therapy, School of Primary and Allied Health Care, Monash University, Melbourne, Victoria.; f National Centre for Healthy Ageing, Victoria.; g Lived Experience Research Partner, Sydney, New South Wales.; h Lived Experience Research Partner, Melbourne, Victoria.; i Department of Neuroscience, Monash University, Melbourne, Victoria.; j Lived Experience Research Partner, Kent.; k Occupational Therapy Department, Alfred Health, Melbourne, Victoria.

**Keywords:** Consumer participation, Motor skills, Outcome assessment, health care, Rehabilitation, Stroke, Stroke rehabilitation, Upper extremity

## Abstract

**Objective:**

To identify which upper-extremity outcome measures stroke survivors and caregivers consider meaningful and acceptable.

**Design:**

Cross-sectional online survey.

**Setting:**

Community-based survey distributed through stroke organizations and social media.

**Participants:**

Thirty respondents (27 stroke survivors, 3 caregivers); median age 64 years (interquartile range=18); median 3.1 years post stroke (interquartile range=6.1).

**Interventions:**

Not applicable.

**Main Outcome Measures:**

Participants rated their familiarity with, perceived relevance of, and preferences for 15 outcome assessments used to measure upper-extremity recovery. They also ranked preferences for timing, frequency, location, and duration of assessments in a hypothetical clinical trial.

**Results:**

Participants most valued clinician-assessed impairment measures (eg, range of motion, strength; 76.7%), task-oriented upper-extremity activity measures (70%), and movement analysis using sensors (66.7%). Measures reflecting neurophysiology or biological mechanisms (eg, biomarkers, transcranial magnetic stimulation) were perceived as less meaningful, particularly among participants with limited prior exposure to these measures. The Motor Assessment Scale, movement sensors, and range-of-motion assessments received higher relevance ratings than dexterity measures, such as the Box and Block Test and the 9-Hole Peg Test. In a hypothetical trial, weekly assessments during the intervention phase (63.0%) were preferred, with in-person assessment sessions, either in hospital outpatient clinics or at home, being the most acceptable. The preferred session duration for outcome assessment was 30-60 minutes; assessments with sessions ≥3 hours were the least acceptable.

**Conclusions:**

Stroke survivors and caregivers prioritize upper-extremity impairment and activity-based upper-extremity assessments and prefer frequent, shorter, in‑person evaluations. Incorporating consumer preferences into clinical care and trial design may enhance the relevance, acceptability, and engagement of stroke survivors in future upper‑limb rehabilitation research.

Involving consumers (eg, people with lived experience of stroke and/or their caregivers) in research is widely recognized as best practice.^1^, ^2^, ^3^ Despite growing expectations for consumer involvement, consumers are seldom involved in choosing trial outcome measures, yet these choices shape what is considered meaningful improvement and whether treatments are deemed effective.^1^ In stroke rehabilitation clinical care and trials, outcome assessments of impairment, function, and quality of life are administered to evaluate intervention efficacy.^4^ Contemporary stroke research has placed greater emphasis on standardizing outcome measures in upper-limb rehabilitation trials to support comparisons of results across countries and institutions and to further the field’s understanding of post stroke recovery.^4^ The Stroke Recovery and Rehabilitation Roundtable provide consensus-based recommendations for upper-limb measurement;^4^ however, the alignment of these recommendations with consumers’ priorities remains unclear.^5^ Therefore, the aim of this study was to understand which upper-limb measurement tools stroke survivors and caregivers consider meaningful and acceptable to guide the selection of outcomes for future clinical trials evaluating upper-limb rehabilitation and clinical practice.

## Methods

A cross-sectional online survey design was used.^6^ Ethical approval was obtained in February 2024 from Monash University (project ID: 41443). An electronic Participant Information Sheet was embedded at survey commencement, and consent was implied by participation.

### Participants and recruitment

Participants were aged ≥18 years and either stroke survivors or family members/caregivers. Recruitment occurred through stroke non-government organizations (Stroke Foundation of Australia, Stroke Recovery NSW) and investigators’ professional social media accounts (eg, X and LinkedIn). Study advertisements included a brief study description, an invitation, and a survey link. Participants could optionally enter a prize draw via a separate webpage at the end of the survey. Passive snowball sampling was used, with participants invited to share the survey link with other stroke survivors in their networks.^6^

### Data collection

Hosted on Qualtrics^a^ survey software, the survey consisted of 4 components: (1) demographics and experiences of arm recovery poststroke; (2) alignment of stroke-related outcome areas with participants’ priorities; (3) prior experience with 15 outcome measures (rated as “I have experienced this measure,” “I am not sure,” or “I haven’t experienced this measure”); (4) perceptions of whether these measures captured any meaningful change. Outcome measures were selected for inclusion in the survey if they are commonly used in stroke rehabilitation studies or practice, or are currently included in international consensus recommendations for use in stroke upper-limb trials.^4^^,^^7^^,^^8^ Links were provided to video demonstrations of the included outcome measures to support familiarity with them. Participants also ranked their preferences for the *timing, frequency, location,* and *duration* of outcome assessments in a hypothetical clinical trial. The survey took approximately 10 minutes to complete.

### Data analysis

Completed surveys were exported to SPSS version 28.0^b^ and then analyzed using descriptive statistics (means and proportions).

## Results

Thirty participants completed the survey. Most were stroke survivors (n=27, 90%), with the remaining participants’ caregivers of a person diagnosed with stroke (n=3, 10%). Participants had a median age of 64 years (IQR 18.0) and most were several years after their stroke (median 3.1 years, IQR 6.1 years). Participants reported varied arm recovery, ranging from no recovery to near-complete recovery (table 1).

**Table 1:** Participant demographics (N=30)

Demographics	Total Sample N=30
Stroke survivor, n (%)	27 (90)
Caregiver/family member of someone who has been diagnosed with a stroke, n (%)	3 (10)
Sex	
Women	15 (50)
Men	15 (50)
Years poststroke, median (IQR)	3.13 (6.1)
Age (y), median (IQR)	64.0 (18.0)
Amount of perceived recovery in arm and/or hand after stroke (0-100)*, median (IQR)	45.0 (51.3)
Self-reported everyday arm use in the home and community, n (%)	
No use of the stroke-affected arm	7 (23.3)
Use of the affected side to help stabilize objects only during 2-handed activities	3 (10)
Limited assistance or easy reach	5 (16.7)
Some reach and grasp with hand manipulation	4 (13.3)
Everyday use unless potential negative consequences	8 (26.7)
Full use	3 (10)

Abbreviations: IQR, interquartile range.

⁎ 0=no recovery to 100=full recovery.

Most participants agreed that kinematics using robotics or movement sensors (n=20, 66.7%), impairment assessments (eg, range of motion, strength, spasticity) (n=23, 76.7%), and assessments of common upper-limb activities (eg, reach and grasp) (n=21, 70.0%) reflected their priorities. Measures of biological mechanisms, such as the presence of biomarkers (eg, gene expression via saliva or blood samples [n=8, 26.7%]), and measures of disability and quality of life that were not specific to stroke were more commonly reported as not reflecting consumer priorities (table 2).

**Table 2:** Participant priorities for stroke-related outcome areas (N=30)

Measure	Yes, Reflects My Priorities, n (%)	Undecided, n (%)	No, Doesn’t Reflect My Priorities, n (%)
Brain scans to assess brain changes in the cortical region that controls the hand and arm	14 (46.7)	8 (26.7)	8 (26.7)
Brain activity measures, such as TMS, to show motor signal changes to the hand and arm	13 (43.3)	13 (43.3)	4 (13.3)
Blood or saliva samples to assess for the presence of biomarkers	11 (36.7)	11 (36.7)	8 (26.7)
A neurological score to assess the severity of stroke, rated by a clinician	15 (50.0)	7 (23.3)	8 (26.7)
Movement analysis using robotics or movement sensors	20 (66.7)	6 (20.0)	4 (13.3)
Assessment of impairments in the affected arm and/or hand (such as range of movement, strength, spasticity) rated by a clinician	23 (76.7)	2 (6.7)	5 (16.7)
Assessment of common arm and/or hand activities (such as reach, grasp, turning a key, using a pen) rated by a clinician	21 (70.0)	2 (6.7)	7 (23.3)
A more general assessment of your level of disability, where your overall disability is rated by a clinician	13 (43.3)	7 (23.3)	10 (33.3)
A self-rating of the amount of movement in the arm and hand	19 (63.3)	4 (13.3)	7 (23.3)
A self-rating of your quality of movement in the arm and hand	19 (63.3)	6 (20.0)	5 (16.7)
A self-rating of your ability to perform basic activities of daily living, such as upper body dressing or showering	20 (66.7)	3 (10.0)	7 (23.3)
A self-rating of your ability to perform extended activities of daily living, such as driving or shopping	19 (63.3)	3 (10.0)	8 (26.7)
A self-rating of your quality of life as a result of your arm and/or hand problems	21 (70.0)	2 (6.7)	7 (23.3)
A self-rating of your quality of life, not specific to the health of your hand and arm	17 (56.7)	4 (13.3)	9 (30.0)

Abbreviations: TMS, transcranial magnetic stimulation.

**Fig 1:**
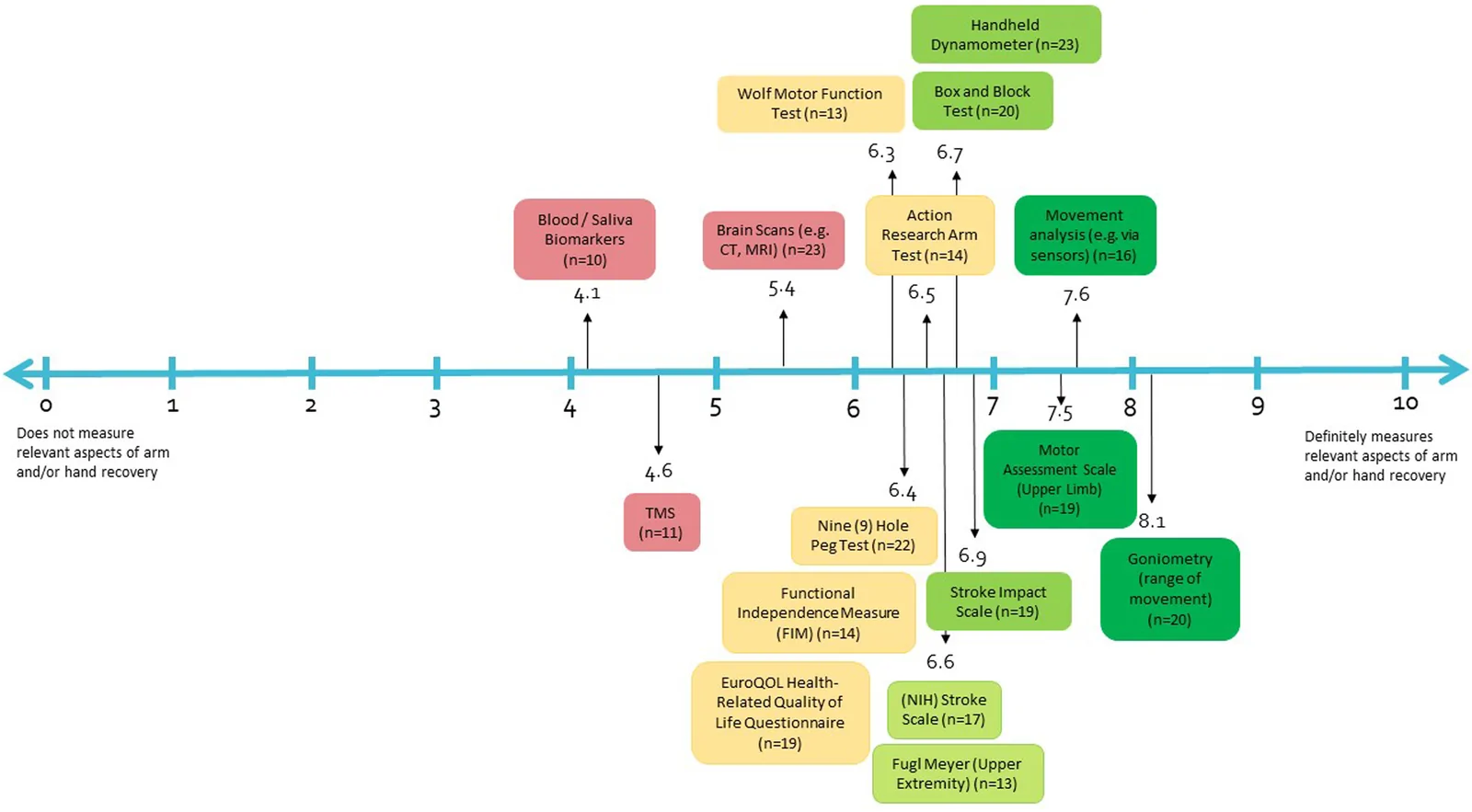
Relevance of commonly used outcome measures to aspects of arm and hand recovery (N=30). CT, computed tomography; MRI, magnetic resonance imaging; NIH, National Institutes of Health; TMS, transcranial magnetic stimulation.

Other than brain imaging with computed tomography or magnetic resonance imaging (n=21, 70%), the most commonly used measures were goniometry (n=20, 66.7%), the Box and Block Test (n=20, 66.7%), the 9-Hole Peg Test (n=19, 63.3%), and grip strength (n=18, 60.0%). However, experience with more advanced or experimental measures was limited. Most participants had not experienced movement sensors (n=21, 70.0%) or biomarkers (n=19, 63.3%). Experience with transcranial magnetic stimulation was mixed, with half of the participants reporting no prior experience (n=15, 50.0%). Of the 15 measures rated for relevance, the highest ratings were for the Motor Assessment Scale (mean ± SD, 7.5±2.65), which incorporates tasks such as picking up a cup, movement analysis using sensors (mean ± SD, 7.6±2.7), and range of movement assessment (mean ± SD, 8.1±2.1). Measures of dexterity, such as the Box and Block Test (mean ± SD, 6.7±3.0) and the 9-Hole Peg Test (mean ± SD, 6.4±3.1), were considered moderately relevant to aspects of arm and hand recovery (fig 1).

Twenty-seven participants responded to the questions about a hypothetical clinical trial. Most preferred weekly assessments during the intervention phase to detect incremental improvement (n=17, 63.0%). The least preferred assessment time point was 1 year post intervention (n=15, 55.6%). Participants preferred in-person assessments, either in a hospital outpatient clinic (n=12, 44.4%) or at home (n=12, 44.4%), over remote assessments. The preferred assessment session duration was 30 minutes (n=9, 33.3%) to 1 hour (n=7, 25.9%), with assessments of ≥3 hours being the least preferred (n=17, 63.0%).

## Discussion

This study identified outcome measures valued by potential trial participants, offering insights into which measures to prioritize when designing future upper-limb rehabilitation trials, as well as considerations for outcome measurement in clinical practice. Stroke survivors most valued clinician-assessed impairment and task-oriented arm and hand assessments, viewing them as directly reflective of their recovery. This finding aligns with research on stroke^5^ and other neurological populations; for example, consumers with cerebral palsy prioritize changes in motor function and in the performance of activities of daily living as meaningful treatment effects.^9^

Neurophysiological or biomarker-based measures were rated as the least meaningful, although many participants had limited experience with these tests. In this brief survey, we did not further explore why this might be the case. Factors such as unfamiliar terminology, perceived burden, or a lack of clear feedback may influence perceptions of usefulness.^5^ Further qualitative research may be needed to understand these views, especially given the role of biomarkers and mechanistic measures in explanatory trials.^8^

Preferences for frequent, shorter assessments of arm and hand function during an intervention phase have important implications for future clinical trial design. Standard trial practice commonly includes only baseline, post intervention, and follow-up time points; however, participants in this study expressed a desire for more granular feedback and monitoring. Incorporating assessments at a higher frequency, balanced against burden, has the potential to improve participant engagement, retention, and satisfaction. Participants also valued in‑person contact with researchers, suggesting that the introduction of remote testing in motor trials and clinical practice, given the rapid expansion of telerehabilitation models,^10^ may require careful explanation to increase acceptability. Additionally, how findings are communicated back to stroke survivors requires consideration, as feedback to participants has been reported to be provided inconsistently.

### Study strengths and limitations

A key strength of this study is its broad mapping of upper-limb trial assessment approaches and their acceptability, providing insights that have not been widely documented. Another strength was the meaningful involvement of consumer investigators as full co-investigators, which contributed to the study’s design, interpretation, and reporting. We acknowledge, however, several limitations. Despite the use of broad recruitment strategies, the sample size was small, which may limit the generalizability of the findings. Funding constraints prevented translation, restricting participation to English-speaking respondents. International perspectives, particularly from settings where stroke rehabilitation models differ, were not captured.

## Conclusions

Stroke survivor and caregiver priorities regarding the types, timing, and frequency of arm and hand outcome measures differ from current research conventions used in upper-extremity rehabilitation trials and clinical practice. The future inclusion of these preferences in study design will help ensure that trial outcomes are meaningful, acceptable, and reflective of what matters most to people with lived experience of stroke.

## Suppliers

a. Qualtrics XM; Qualtrics.
b. SPSS, version 28; IBM.

## Disclosure

Supported by the Heart Foundation of Australia (grant no.106762) (N.A.L). The investigators have no non-financial disclosures to make in relation to this project.
